# P-825. Clinical Features and Antimicrobial Susceptibility Patterns of Vancomycin-Resistant Enterococcus faecalis: A Single-Center Retrospective Study

**DOI:** 10.1093/ofid/ofaf695.1033

**Published:** 2026-01-11

**Authors:** Samantha Aguilar, Mollie VanNatta, Aline Arif, Destyn Dicharry, Alexandre E Malek

**Affiliations:** Ochsner LSU Health Shreveport, Shreveport, LA; Ochsner LSU Health Shreveport, Shreveport, LA; LSU Health Shreveport, Shreveport, Louisiana; Louisiana State University Health Shreveport, Medical School, Shreveport, Louisiana; LSU Health Shreveport, Shreveport, Louisiana

## Abstract

**Background:**

Despite its frequent susceptibility to ampicillin (Amp), vancomycin-resistant *Enterococcus faecalis* (VRE) is often clinically managed with Datpomcyin (Dapto) or other novel antibiotics. Broad-spectrum antibacterial agents raised concerns about overtreatment and challenged antimicrobial stewardship programs. This clinical study aims to assess the local *E. faecalis* VRE isolates susceptibility patterns and evaluate antibiotic treatments.

**Table 1.**
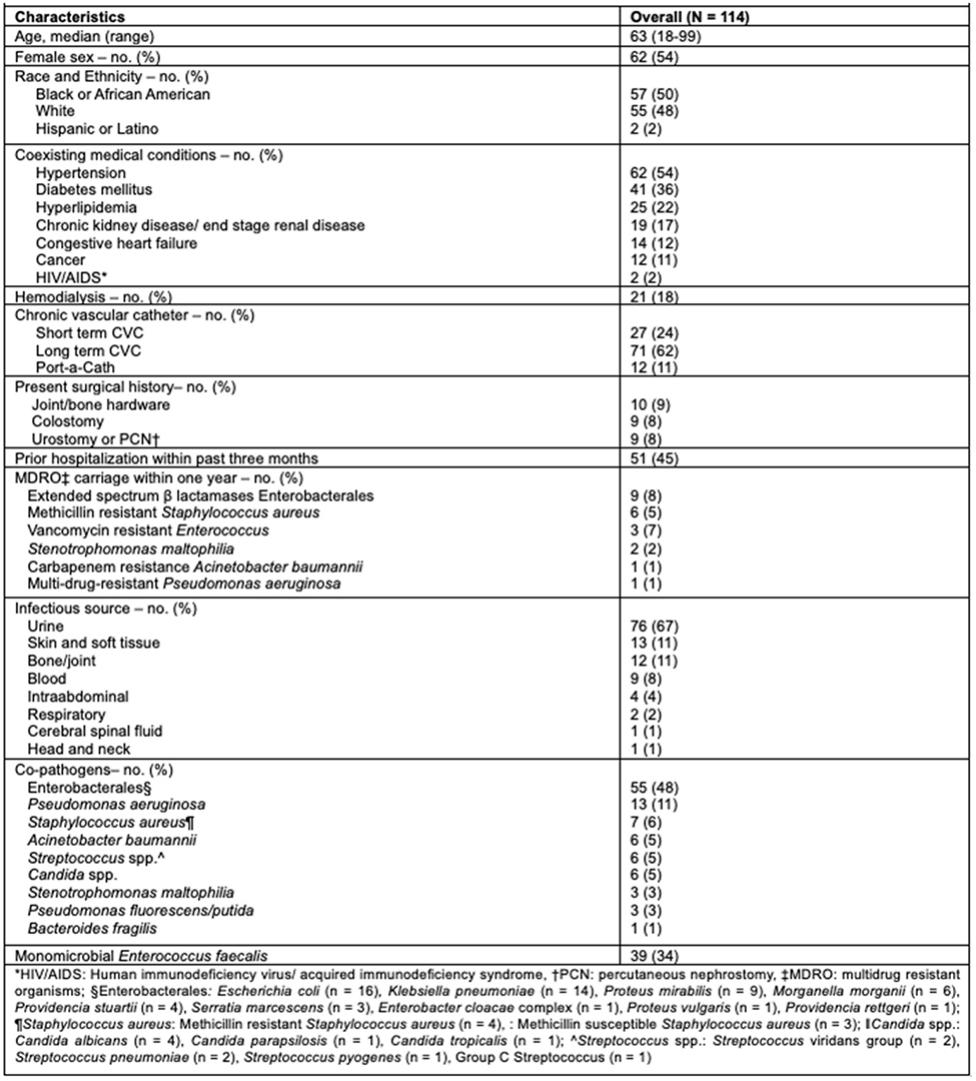
Characteristics of the Patients, Infections, and Co-pathogens

**Table 2.**
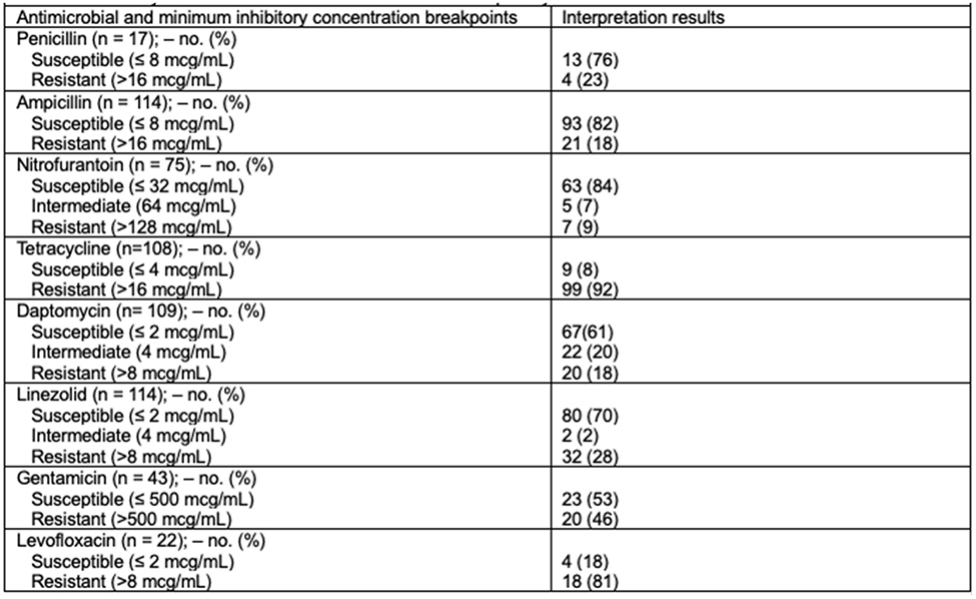
Vancomycin Resistant Enterococcus faecalis Susceptibility Pattern

**Methods:**

We conducted a retrospective cohort study of adult patients who were admitted at Ochsner LSU Health - Academic Medical Center between June 2018 and March 2025. We excluded Pts with concomitant non-*Enterococcus faecalis* VRE infections and those aged ≤18 years. Vancomycin resistance was defined by a minimum inhibitory concentration of >32 mcg/mL according to the 35^th^ edition of the Clinical and Laboratory Standards Institution breakpoints.

**Table 3.**
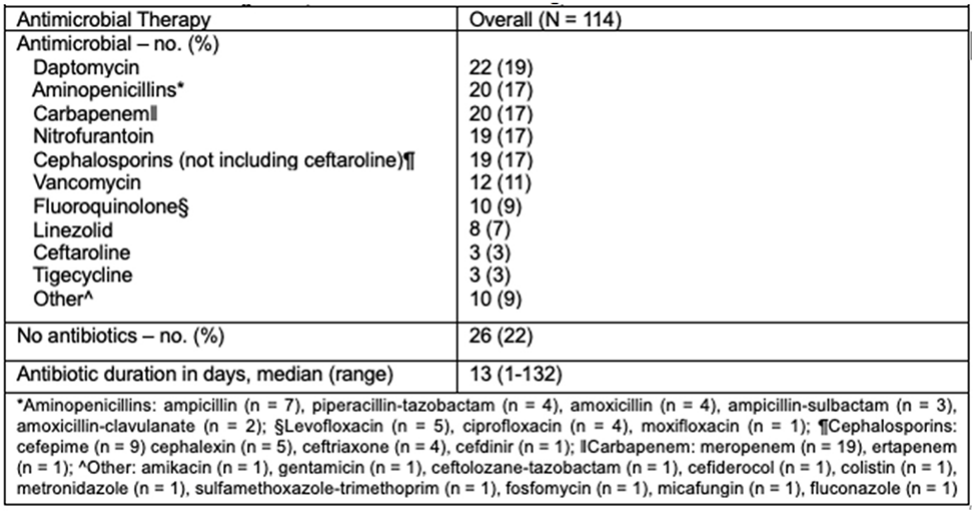
Features of Antimicrobial Regimens

**Table 4.**
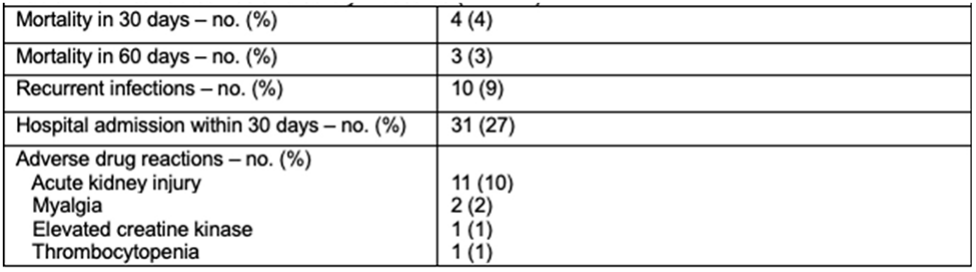
Clinical Outcomes and Reported Adverse Events

**Results:**

A total of 337 patients were screened, 114 pts met the inclusion criteria. Baseline characteristics are summarized in Table 1. The cohort was 54% female and 50% AA, with 54% having HTN, 36% DM, and 19% of Pts had a history of MDRO carriage. The most common source of infection was urinary tract (67%), followed by SSTIs and bone/joint infections (11% each). The most frequently identified co-pathogens were *Enterobacterales* (48%). Resistance profiles are listed in Table 2. Out of 114 isolates, 82% were susceptible to Amp, 84% to nitrofurantoin, 61% to Dapto, and 70% to linezolid. Antimicrobial regimens are detailed in Table 3, with Dapto being the most used agent (19%). Clinical outcomes are presented in Table 4. Within 60 days, 7% of patients died, 9% experienced reinfection to the same pathogen, and 27% of pts were readmitted within 30 days. Adverse drug reactions were infrequent, with AKI occurring in 10% of pts. There were no statistically significant differences in 30-day mortality or relapse infections between pts who received an Amp-based regimen vs pts who were treated with non-Amp-based antibiotics.

**Conclusion:**

Despite higher susceptibility to ampicillin and nitrofurantoin, daptomycin was the most used agent for *Enterococcus faecalis* VRE infections. These findings highlight an unmet need for improved antimicrobial stewardship and further clinical studies to guide optimal therapy.

**Disclosures:**

All Authors: No reported disclosures

